# **Association of non**-**synonymous single nucleotide polymorphisms in the *LOXL1* gene with pseudoexfoliation syndrome in India**

**Published:** 2008-02-09

**Authors:** Vedam Lakshmi Ramprasad, Ronnie George, Nagasamy Soumittra, Ferdinamarie Sharmila, Lingam Vijaya, Govindasamy Kumaramanickavel

**Affiliations:** 1 Department of Genetics and Molecular Biology, Vision Research Foundation; 2 Department of Glaucoma, Medical Research Foundation, Sankara Nethralaya, Chennai, India

## Abstract

**Purpose:**

In the Icelandic and Swedish populations, pseudoexfoliation syndrome (XFS) and pseudoexfoliation glaucoma (XFG) has been significantly associated with *LOXL1* exon 1 polymorphisms - allele G of rs1048661 (R141L) and allele G of rs3825942 (G135D). In this study, we looked at the association of rs1048661 and rs3825942 in a southern Indian population.

**Methods:**

Fifty-two cases with XFS (including XFG) and 97 matched controls that had thorough glaucoma evaluations were included in the study. Exon 1 of the *LOXL1* gene with the single nucleotide polymorphisms (SNPs) were amplified and sequenced. For statistical significance, Pearson’s Χ^2^ test was performed. The HAPLOVIEW program v4.0 was used to determine the Hardy–Weinberg equilibrium and haplotype association.

**Results:**

In our study population, there was a significant association of allele G of rs3825942 with XFS (p=0.0001) and genotype GG (p=0.000305) with XFS.

**Conclusions:**

Out of the two non-synonymous SNPs in exon 1 of the *LOXL1* gene, rs3825942 has a significant association with XFS cases in the patients of the southern Indian population. To the best of our knowledge, this is the first Asian study replicating the European studies.

## Introduction

Pseudoexfoliation syndrome (XFS) is an age-related, systemic, elastic microfibrillopathy affecting both intraocular and extraocular tissues [1]. In the eye, XFS is characterized by the presence of fibrogranular extracellular debris in the anterior segment, which is composed of a complex glycoprotein–proteoglycan structure having epitopes of a basement membrane, elastic fiber system, and components of elastic microfibrils [2]. The average worldwide prevalence of XFS is 10%–20% of the general population over the age of 60 years. However, studies have shown much higher prevalence in certain populations like Scandinavian countries and Greece [3-5].

Glaucoma, the second most common cause of blindness, is a heterogeneous group of disorders characterized by optic nerve damage [6]. XFS is the most common identifiable cause of secondary glaucoma such as pseudoexfoliation glaucoma (XFG), which is due to the accumulation of the exfoliative material [7]. The risk of XFS converting to XFG over a period of 15 years is about 60% [8,9].

Linkage studies on a large Finnish family showing an autosomal dominant mode of inheritance for XFS trait identified a locus, 18q12.1–21.33, with a two point LOD score of 3.45 and a multipoint LOD score of 4.2 and few other loci like 2q, 17p, and 19q previously linked to primary open angle glaucoma [2]. Familial aggregation and the increased frequency of XFS and glaucoma in relatives of affected subjects compared with relatives of unaffected subjects clearly suggests an underlying genetic component [10,11].

A recent genome wide association study on Icelandic patients identified three single nucleotide polymorphisms (SNPs) on chromosome 15q24.1 to be significantly associated with XFS and XFG.

The three SNPs are, allele T of rs2165241 in the first intron and allele G of rs1048661 (R141L) and allele G of rs3825942 (G135D) in exon 1 *LOXL1*. The association was further confirmed in a Swedish population, and the combined p values of the SNPs were 1.0x10^-27^ (rs2165241), 2.3x10^-12^ (rs1048661) and 3.0x10^-21^ (rs3825942) [9]. *LOXL1* gene expression is detected in ocular tissues, and it is a member of the lysyl oxidase family of proteins [12]. This protein is involved in catalyzing the deamination of lysine residues of tropoelastin, causing cross-linking and the formation of elastin polymer fibers [13,14]. The pathological process of XFS is characterized by chronic accumulation of abnormal fibrillar material in the anterior segment of the eye, leading to numerous clinical complications apart from secondary glaucoma [7]. Though the precise role of *LOXL1* in the XFS is not known, it could possibly contribute to the pathogenesis due to the accumulation of abnormal elastin microfibrillar components.

In this study, we have genotyped the two non-synonymous SNPs, rs1048661 and rs3825942, in the XFS and compared it with normal controls in a southern Indian population.

## Methods

### Cases and controls

Fifty-two cases of XFS with or without glaucoma or ocular hypertension and 97 matched healthy controls were taken for the study. Cases and controls were matched for age, gender, and ethnicity. Subjects and controls were recruited from the glaucoma clinic at the hospital and also from a population-based survey that was being conducted at the Sankara Nethralaya Eye Hospital in Chennai, India after the approval of the local research ethics committee. All subjects and controls underwent a comprehensive ocular examination including vision and refraction, slit lamp assessment, applanation tonometry, gonioscopy, and dilated stereobiomicroscopic optic disc and retinal examination. Presence of pseudoexfoliation was diagnosed if the pseudoexfoliative material was detected on the pupillary ruff or anterior lens capsule.

Only bilaterally phakic subjects were enrolled as controls. Blood samples were collected from the cases and controls after obtaining an informed consent, and the study procedures were performed in accordance with Institutional Review Board guidelines and the Declaration of Helsinki.

### Polymerase chain reaction and sequencing

The region of the *LOXL1* gene harboring the SNPs, rs1048661 (R141L) G and rs3825942 (G153D) G, was amplified and sequenced to genotype the SNPs. The fragment containing the SNPs were amplified using the primers, 5′ ATT CGG CTT TGG CCA GGT 3′ and 5′ GAA CTG CTG CGG GTA GGA 3′, in a 20 μl reaction containing 10 mM Tris (pH 9.0), 50 mM KCl, 1.5 mM MgCl_2_ and 0.01% gelatin, 1 mM dNTP each (GeNei, Bangalore, India), 10 μM of each forward and reverse primer, 1 U of Taq DNA polymerase (GeNei, Bangalore, India), 5 mM betaine (Sigma Aldrich, St. Louis, MO), and 100 ng of genomic DNA.

The polymerase chain reaction (PCR) protocol was as follows: initial denaturation at 94 °C for 5 min followed by denaturation at 94 °C for 30 s, annealing at 58–55 °C touchdown for 30 s with 0.5 °C decrement for the first six cycles, extension at 72 °C for 45 s for 35 cycles, and a final extension of 72 °C for 5 min. PCR products were digested with exonuclease I and *E.coli* and shrimp alkaline phosphatase (Fermentas, Life Sciences, Glen Burnie, MD). The purified PCR products were sequenced unidirectionally using BigDye Terminator v.3.1 kit (Applied Biosystems, Foster City, CA) with specific primers in ABI3100 Avant (Applied Biosystems). The sequences were analyzed in Sequence Analysis software v.3.1.1.

### Statistical analysis

Statistical analysis was performed using SPSS# version 14.0. Those with pseudoexfoliation (irrespective of the presence or absence of glaucoma or ocular hypertension) were compared to the control population. Pearson’s Χ^2^ test (adjusted by Yates correction where necessary) was used to compare patient and control groups for possible associations between SNP allele frequency/haplotype frequency and disease state. Bonferroni correction for multiple tests was applied and a p value of less than 0.00625 was considered significant. Odds ratios were also calculated. Linkage disequilibrium analysis and checking of conformance of Hardy–Weinberg equilibrium were done using the HAPLOVIEW program (version 4.0). It calculates several pair-wise measures of linkage disequilibrium (LD). Individual haplotypes and their estimated population frequencies were inferred using the HAPLOVIEW program with all parameters set at the default values. The genotype data were loaded as unphased diplotypes in a standard linkage format. Marker information including the name and physical location was loaded separately.

## Results

A total of 52 cases (22 XFS, 11 XFS + ocular hypertension [OHT], and 19 XFG) and 97 unrelated controls all of south Indian ethnicity were studied. Cases ranged in age from 45 to 107 with an average age of 68.9 (standard deviation: 11.43) years. The controls were in the age range of 46 to 84 with an average age of 64.1 (SD: 7.2) years. Among the cases, there were 27 males and 25 females, and among the controls, there were 52 males and 45 females. Summary of the clinical features of patients is given in Table 1.

**Table 1:** Clinical characteristics of subjects with pseudoexfoliation syndrome.

	**Intraocular pressure mean (SD)**	VCDR* Median (SD)
Pseudoexfoliation with glaucoma	23.4 (9.79)	0.81 (0.13)
Psedoexfoliation with ocular hypertension	24.1 (5.17)	0.6 (0.16)
Pseudoexfoliation	14.3 (2.62)	0.5 (0.27)

The asterisk indicates the vertical cup to disc ratio. In bilateral cases, the eye with greater optic disc damage (XFG) or IOP (ocular hypertension or XFS) has been included. None of the patients were on medication at presentation.

Between the two non-synonymous SNPs, rs1048661 did not show an association with XFS (p=0.156 for allele G, odds ratio (OR)=1.49, confidence interval (CI) – 0.89 −2.51), whereas allele G of rs3825942 showed a significant association (p=0.0001, OR=4.17, CI – 1.89–9.18). Case-control ratios of associated allele G of rs1048661 and rs3825942 are shown in Table 2. Genotype GG of the rs1048661 was also not significantly associated with XFS (p=0.058), and similar to the allelic association, genotype GG of rs3825942 was significantly associated with XFS (p=0.000305; Table 2).

**Table 2:** *LOXL1* allele/genotype count and frequencies in cases and controls.

**SNP**		**Cases**		**Controls**		**p value**
	Allele/ Genotype	Allele/Genotype count	Allelic/ Genotype frequency	Allele/ Genotype count	Allelic/ Genotype frequency	
R141L (rs1048661)	G	75	0.72	123	0.63	0.156
	T	29	0.27	71	0.36	
G153D (rs3825942)	G	96	0.92	144	0.74	0.0001
	A	8	0.07	50	0.25	
R141L (rs1048661)	GG	29	0.55	36	0.37	0.058
	GT	17	0.32	51	0.52	
	TT	6	0.11	10	0.1	
G153D (rs3825942)	GG	45	0.86	52	0.53	0.000305
	GA	6	0.11	40	0.41	
	AA	1	0.01	5	0.05	

Allelic/genotype association testing results of rs1048661 (R141L) and rs3825942 (G135D) SNPs in exon 1 of *LOXL1* with pseudoexfoliation syndrome (XFS) in Indian cases and controls.

Allelic frequencies of both the SNPs in cases and controls were in Hardy–Weinberg equilibrium. The observed (0.473) and expected (0.443) heterozygosity for rs1048661 did not differ significantly (p=0.55). Similarly, there was no departure in the observed (0.311) and expected (0.315) heterozygosity for rs3825942 (p=1).

The two SNPs are in linkage disequilibrium (D’=1), and as observed in the Icelandic study, only three of the four possible haplotypes were detected in our samples.

Further comparing the haplotype results, the (G, G) had an OR of 5.73 (p<0.0001, CI – 2.534, 12.98) and the (T, G) had an OR of 2.55 (CI – 1.08, 6.05, p=0.0476) relative to (G, A). Relative to (T, G), the (G, G) had an OR of 2.22 (p=0.0006, CI −1.28, 3.82) and (G, A) had an OR of 0.21 (p=0.0002, CI −0.093, 0.49). Among the three observed haplotypes, (G, A) has a protective effect.

We did not find any other polymorphisms either in the cases or controls except for the SNPs mentioned earlier in exon 1 of the gene.

## Discussion

XFS is an age-related, systemic, elastic microfibrillopathy characterized by the production of abnormal basement membrane-like material in several intraocular and extraocular tissues. It is the most common cause of secondary open angle glaucoma worldwide [1].

Genetic and environmental factors have been implicated in the pathogenesis of XFS. In a recent breakthrough genome-wide association study done in an Icelandic population, which was replicated in a Swedish population, the genetic etiology of XFS and its associated glaucoma was unraveled. Polymorphisms in the coding region of the *LOXL1* gene located on chromosome 15q24.1 are strongly associated with XFS and XFG in these populations [9]. The Thorleifsson et al. [9] study have shown a strong association of XFS and XFG with two known non-synonymous SNPs, rs1048661 (Arg→Leu, R141L) and rs3825942 (Gly→Leu, G135D), of the *LOXL1* gene.

In the present study, we have identified 52 cases (22 XFS, 11 XFS+OHT, and 19 XFG) and 97 unrelated controls of south Indian ethnicity. In the identified cases and controls, we genotyped the two non-synonymous SNPs, rs1048661 (Arg→Leu, R141L) and rs3825942 (Gly→Leu, G135D), which are located in exon 1 of the *LOXL1* gene.

When compared to the Icelandic study, we did not find significant association with rs1048661, but we replicated the significant association of allele G of SNP, rs3825942 (p=0.0001, OR=4.17, CI – 1.89–9.18). It is interesting that Thorleifsson et al. [9] demonstrated that the expression of *LOXL1* was reduced by an estimated 7.7% with each copy carrying the risk G allele of rs1048661. The lack of association seen for the G allele of rs1048661 in the present study may be attributed to the fact that the sample size was small. Similar to the Icelandic study, only three of the four possible haplotypes were detected in our samples. Among the three observed haplotypes, (G,G) was strongly associated with XFS (OR of 3.001, CI – 1.83, 4.91, p=9.9 × 10^−6^). (G,A) has a significant protective effect (p=2.0 × 10^−4^) as it is seen more in controls (Table 3).

**Table 3:** Results of haplotype association testing.

rs1048661 ** - rs3825942 Haplotype**	**Cases**	**Frequency**	**Controls**	**Frequency**	**Chi square**	**p value**
GG	67	0.64	73	0.37	19.51	9.9 × 10-6
TG	29	0.27	71	0.36	2.305	0.1289
GA	8	0.07	50	0.25	14.121	2.0 × 10-4

Haplotype association testing results for the 2 tagging SNPs of rs1048661 (R141L) and rs3825942 (G135D) in exon 1 of *LOXL1* gene with pseudoexfoliation syndrome (XFS) in Indian cases and controls. Haplotypes composed of the two *LOXL1* SNPs were determined using HAPLOVIEW program (version 4.0). This table shows the Χ^2^ and p values (corrected for multiple testing bias) permuted by HAPLOVIEW by comparing one haplotype with the other two between cases and controls.

The *LOXL1* gene is a member of the lysyl oxidase gene family. The prototypic member of the family is essential for the biogenesis of connective tissue and encodes for an extracellular copper-dependent amine oxidase that catalyzes the first step in the formation of cross-links in collagens and elastin [15]. Lysyl oxidases (LOX) and lysyl oxidase-like-proteins form an enzyme family with diverse NH_2_-termini and conserved COOH-terminal lysyl oxidase (LOX) domains. A highly conserved amino acid sequence at the COOH-terminus end appears to be sufficient for amine-oxidase activity, indicating that each member of the protein family may have this particular function [16].

In addition to LOX and LOXL1, three closely related LOX-like proteins were identified in mammals in the past decade, LOXL2, LOXL3, and LOXL4 [16]. Although the LOX family members share some homology, each of the genes are located on different chromosomes; *LOX* is located on chromosome 5q23.1, *LOXL1* on chromosome 15q24.1, *LOXL2* on chromosome 8p21.3, *LOXL3* on chromosome 2p13.1, and *LOXL4* on chromosome 10q24.2 as per the human genome data at Ensembl.

In the case of age-related macular degeneration (AMD), the identification and validation of the association between the *CFH* gene variants and AMD [17] made many investigators look at other genes in the complement factor H related family (*CFHR*) genes like complement factor B (*BF*) and complement component 2 (*C2*) [18]. Further work revealed the strong association of *CFHR* genes with AMD. Similarly, it would be worth hypothesizing that other LOX family member genes (*LOX, LOXL2, LOXL3, *and *LOXL4*) could be strong candidates whose variants might be associated with XFS and/or XFG. The prevalence of pseudoexfoliation syndrome in India has been reported to be between 3% and 6% in those aged 40 years and above [19-21]. XFS is strongly associated with not only cataract and complications of cataract surgery but also open angle glaucoma, and it shows an increasing prevalence with age [22]. Pseudoexfoliation was associated with a significantly greater risk of blindness secondary to these causes. Hence, this finding gains importance and has public health implications for India.

In summary, our results showed that of the two non-synonymous SNPs in exon 1 of the *LOXL1* gene that were shown to be significantly associated with XFS, rs3825942 (G135D) conferred a significant risk to develop the disease in the Indian population. To the best of our knowledge, this is the first study showing an association of *LOXL1* gene polymorphism and XFS in the Asian population.
